# The SGLT2 inhibitor checklist: a comprehensive review of perioperative and acute phase safety management

**DOI:** 10.3389/fendo.2026.1777334

**Published:** 2026-02-11

**Authors:** Bin Deng, Wenhua Liu, Qingmin Chu

**Affiliations:** 1 Department of Cardiology, Shenzhen Bao’an Chinese Medicine Hospital, Shenzhen, China; 2 Department of Endocrinology, Bao’an Authentic TCM Therapy Hospital, Shenzhen, China; 3 Department of Cardiology, The First Affiliated Hospital of Guangzhou University of Chinese Medicine, Guangzhou, China

**Keywords:** arginine vasopressin (AVP) axis, euglycemic diabetic ketoacidosis (euDKA), ketogenesis, perioperative management, risk stratification, SGLT2 inhibitors, sodium-monocarboxylate transporters (SMCT)

## Abstract

Sodium-glucose cotransporter-2 (SGLT2) inhibitors have transcended their initial designation as mere glucose-lowering agents to become a foundational pillar in the management of cardiorenal metabolic syndrome. Their cardiorenal benefits have made them ubiquitous in surgical patients, including those without diabetes. However, this therapeutic triumph has introduced a unique and deceptive perioperative challenge: euglycemic diabetic ketoacidosis (euDKA). This metabolic emergency, characterized by severe metabolic acidosis and ketosis in the absence of significant hyperglycemia, poses a diagnostic dilemma that continues to jeopardize patient safety. This comprehensive review synthesizes the rapidly evolving landscape of perioperative SGLT2 inhibitor management as of 2025. We provide an exhaustive dissection of the endocrine mechanisms driving ketogenesis, challenging the canonical “insulin-deficiency” model by integrating novel data on human pancreatic α-cell SGLT1 expression, renal sodium-monocarboxylate transporter (SMCT) upregulation, and the newly elucidated arginine vasopressin (AVP)-V1b receptor axis which mechanistically links dehydration to hyperglucagonemia. Furthermore, we critically adjudicate the conflicting clinical evidence emerging in 2024-2025, juxtaposing the reassuring “natural experiment” data from JAMA Surgery against the persistent safety signals in BMJ Open and anesthesia literature. We analyze the risks of “rebound” heart failure associated with medication withdrawal—citing specific hazard ratios for readmission—and provide a comparative analysis of divergent international guidelines (FDA, EMA, ANZCA, JBDS). Finally, we propose a physiologically grounded, risk-stratified clinical decision framework to guide the perioperative suspension and resumption of these potent agents.

## 1 Introduction: the pleiotropic revolution and the perioperative paradox

### 1.1 The therapeutic revolution

The introduction of SGLT2 inhibitors—canagliflozin, dapagliflozin, empagliflozin, and ertugliflozin—marked a watershed moment in metabolic medicine. By inhibiting the high-capacity, low-affinity glucose transporter in the S1 and S2 segments of the proximal renal tubule, these agents promote glycosuria, thereby lowering plasma glucose, weight, and blood pressure independent of insulin secretion (1). However, the narrative of SGLT2 inhibitors has rapidly shifted from glucocentricity to organ protection. Large cardiovascular outcome trials (CVOTs) such as EMPA-REG OUTCOME, CANVAS, and DECLARE-TIMI 58, followed by dedicated heart failure and renal trials (DAPA-HF, EMPEROR-Reduced, DAPA-CKD), have established these drugs as disease-modifying therapies for heart failure and proteinuric kidney disease (2).

### 1.2 The perioperative risk landscape

As a consequence of these expanded indications, the demographic of patients presenting for surgery while on SGLT2 inhibitors has broadened significantly. It now includes not only patients with type 2 diabetes (T2DM) but also those with heart failure or CKD who may not have diabetes, or who have “pre-diabetes.” This expansion complicates the perioperative landscape. The surgical stress response is inherently catabolic, characterized by a surge in counter-regulatory hormones—cortisol, catecholamines, glucagon, and growth hormone—that antagonize insulin and mobilize free fatty acids (3). When this physiology is superimposed on the distinct metabolic milieu induced by SGLT2 inhibition—specifically, a lowered threshold for ketogenesis and a decoupling of glucose levels from insulin signaling—the risk of euDKA emerges.

EuDKA is a particularly insidious complication because it lacks the cardinal warning sign of classic DKA: hyperglycemia. Patients often present with blood glucose levels below 200 mg/dL (11.1 mmol/L), leading clinicians to attribute symptoms of nausea, vomiting, or tachycardia to postoperative ileus or pain rather than a life-threatening metabolic acidosis (4). The mortality and morbidity associated with missed euDKA are non-trivial, involving prolonged intensive care admissions and potential cardiac instability (5).

This review aims to address the pressing need for a nuanced, expert-level understanding of this phenomenon. We move beyond the superficial “stop dates” to explore why these drugs cause ketosis, how new endocrine pathways (like the AVP axis) are reshaping our risk models, and when the benefits of continuation might outweigh the risks of withdrawal.

## 2 Deepening the endocrine mechanisms of SGLT2i-associated ketosis

To master the perioperative management of SGLT2 inhibitors, one must first deconstruct the molecular machinery that predisposes these patients to ketosis. The prevailing view has long been that SGLT2 inhibitor-associated DKA is driven simply by the reduction in insulin dose (due to lower glucose) and a compensatory rise in glucagon. While true, this model is incomplete. Research published through 2024 and 2025 has unveiled a far more complex multi-organ crosstalk involving the pancreatic α-cell, the posterior pituitary, and the renal tubule.

### 2.1 The Pancreatic α-cell controversy: SGLT2 vs. SGLT1

A defining biochemical footprint of SGLT2 inhibition is hyperglucagonemia. Treatment with gliflozins consistently raises plasma glucagon levels, which lowers the insulin:glucagon ratio and drives the liver to switch from glucose oxidation to fatty acid oxidation and ketogenesis (6). The precise mechanism by which SGLT2 inhibitors stimulate the α-cell has been a subject of intense controversy in the endocrine literature.

#### 2.1.1 The “direct effect” hypothesis and its refutation

Initially, it was hypothesized that SGLT2 transporters were present on the surface of pancreatic α-cells and acted as glucose sensors. The theory posited that pharmacological inhibition of these transporters mimicked a state of intracellular “perceived hypoglycemia,” triggering the release of glucagon even when systemic glucose levels were normal (7). However, rigorous molecular interrogation has largely dismantled this hypothesis. As detailed in comprehensive transcriptomic and immunodetection studies (8), SGLT2 expression is virtually undetectable in human and rodent α-cells. Furthermore, functional experiments using isolated islets from humans (29 donors) and rodents demonstrated that direct application of dapagliflozin, empagliflozin, or sotagliflozin did not stimulate glucagon secretion *in vitro (*8).

#### 2.1.2 The emerging role of SGLT1 in humans

While SGLT2 is absent, there is growing consensus regarding the presence and functional importance of SGLT1 (the primary intestinal glucose transporter) in human pancreatic α-cells (8). It is crucial to distinguish this from rodent models, where SGLT1 expression in α-cells is negligible or absent.

- Mechanism: SGLT1 is an electrogenic transporter that couples the uptake of glucose with sodium (Na^+^), utilizing the sodium gradient maintained by the Na^+^/K^+^-ATPase. In the context of the human α-cell, SGLT1 activity appears to be crucial for glucose sensing (9, 10).
- The Paradox: Some studies suggest that under hyperglycemic conditions, SGLT1-mediated sodium influx leads to membrane depolarization or altered intracellular signaling (such as changes in ATP production or cellular acidification), which can dysregulate glucagon secretion (11).
- Clinical Implication: The clinical relevance of this SGLT1 distinction remains nuanced. While preclinical data suggest differential effects on glucagon secretion, this has not consistently translated into a measurably different risk profile for perioperative euDKA in human studies (6). The predominant driver of ketosis appears to be the systemic metabolic shifts induced by SGLT2 inhibition—glycosuria, calorie loss, and dehydration—which are class-wide effects (6, 12). Consequently, current perioperative guidelines do not stratify management based on an agent’s SGLT1 inhibitory potential (13, 14). For the clinician, the practical implication is that the vigilance and management protocols for euDKA should be applied uniformly to all patients on any SGLT2 inhibitor, regardless of SGLT1 selectivity (13). The key takeaway is the shared risk mechanism, not a differential risk based on this specific pharmacological property (15, 16).

### 2.2 The Arginine Vasopressin- glucagon axis

Perhaps the most groundbreaking mechanistic update relevant to the perioperative period is the elucidation of the Arginine Vasopressin (AVP) pathway (17). This axis provides the “missing link” explaining why dehydration is such a potent trigger for euDKA.

#### 2.2.1 The dehydration signal

SGLT2 inhibitors induce osmotic diuresis, increasing urine volume and potentially leading to subclinical or overt hypovolemia and hyperosmolality. In the perioperative setting, this is compounded by preoperative fasting (NPO status) and surgical fluid losses. The physiological response to hyperosmolality is the secretion of AVP (antidiuretic hormone) from the magnocellular neurons of the hypothalamus/posterior pituitary (17).

#### 2.2.2 The V1b receptor connection

Recent high-impact studies have identified that pancreatic α-cells express high levels of the Vasopressin 1b receptor (V1bR, gene Avpr1b) (17–19).

- The Pathway:
  - Stimulus: Dehydration or cellular glucopenia triggers AVP release.
  - Reception: AVP binds to the Gq-protein coupled V1b receptors on the α-cell surface.
  - Signaling: This activates the phospholipase C pathway, leading to increased intracellular calcium (Ca^2+^) mobilization.
  - Effect: The calcium surge triggers the exocytosis of glucagon granules.
- Experimental Validation: In *in vivo* models, dapagliflozin treatment increased plasma copetin (a surrogate for AVP) and glucagon. Crucially, the glucagon-stimulating effect of dapagliflozin was abolished by pharmacological blockade or genetic knockout of the V1b receptor (14).
- Perioperative Significance: This mechanism fundamentally reframes our understanding of euDKA risk. A patient who is fasting and dehydrated is not merely volume-depleted; they are actively driving a potent hormonal signal (AVP) that directly forces the pancreas to pump out glucagon. This AVP-mediated hyperglucagonemia creates a “feed-forward” loop for ketogenesis. It underscores that aggressive fluid management is not just supportive care but a targeted therapy to suppress the ketogenic drive by reducing AVP secretion.

### 2.3 Renal handling of ketones: the “thrifty substrate” and SMCTs

The kidney’s role in euDKA extends beyond being the target of SGLT2 inhibitors; it is also a critical regulator of ketone clearance. Under normal physiology, ketone bodies (acetoacetate and β-hydroxybutyrate) are freely filtered at the glomerulus and reabsorbed in the proximal tubule to conserve energy (20).

#### 2.3.1 SMCT upregulation

The reabsorption of ketones is mediated by Sodium-Monocarboxylate Transporters (SMCT1 and SMCT2, encoded by SLC5A8 and SLC5A12), which are sodium-dependent cotransporters located on the apical membrane of the renal tubule (21).

- Mechanism: These transporters use the sodium gradient to reclaim monocarboxylates (lactate, ketones) from the urine.
- SGLT2i Effect: Research indicates that SGLT2 inhibition, by altering the sodium flux and intracellular metabolic state of the proximal tubule cells, leads to a compensatory upregulation of SMCT1 and SMCT2 expression (21). This phenomenon is part of the “thrifty substrate” hypothesis—the body, perceiving a loss of glucose, attempts to avidly conserve alternative fuels like ketones.
- The Consequence: In a patient developing euDKA, plasma ketone production is high (hepatic origin). Paradoxically, the kidney—primed by SGLT2 inhibitors—increases its reabsorption of these ketones rather than excreting them to lower plasma levels. This mechanism maintains hyperketonemia and explains why urine ketone testing can sometimes be unreliable or lag behind serum levels; the kidney is actively trying to keep the ketones in the blood.

### 2.4 Synthesis: the multi-organ crosstalk of euDKA

The interplay of these mechanisms creates a perfect metabolic storm in the perioperative period.

1. Renal SGLT2 Blockade causes obligate glycosuria and calorie loss.
2. Osmotic Diuresis leads to dehydration, stimulating Pituitary AVP release.
3. AVP binds to Pancreatic V1b Receptors, driving Glucagon secretion.
4. Low Insulin/High Glucagon drives Hepatic Ketogenesis.
5. Renal SMCT Upregulation prevents ketone excretion, trapping ketones in the plasma (“Thrifty Substrate”).

**Figure 1:**
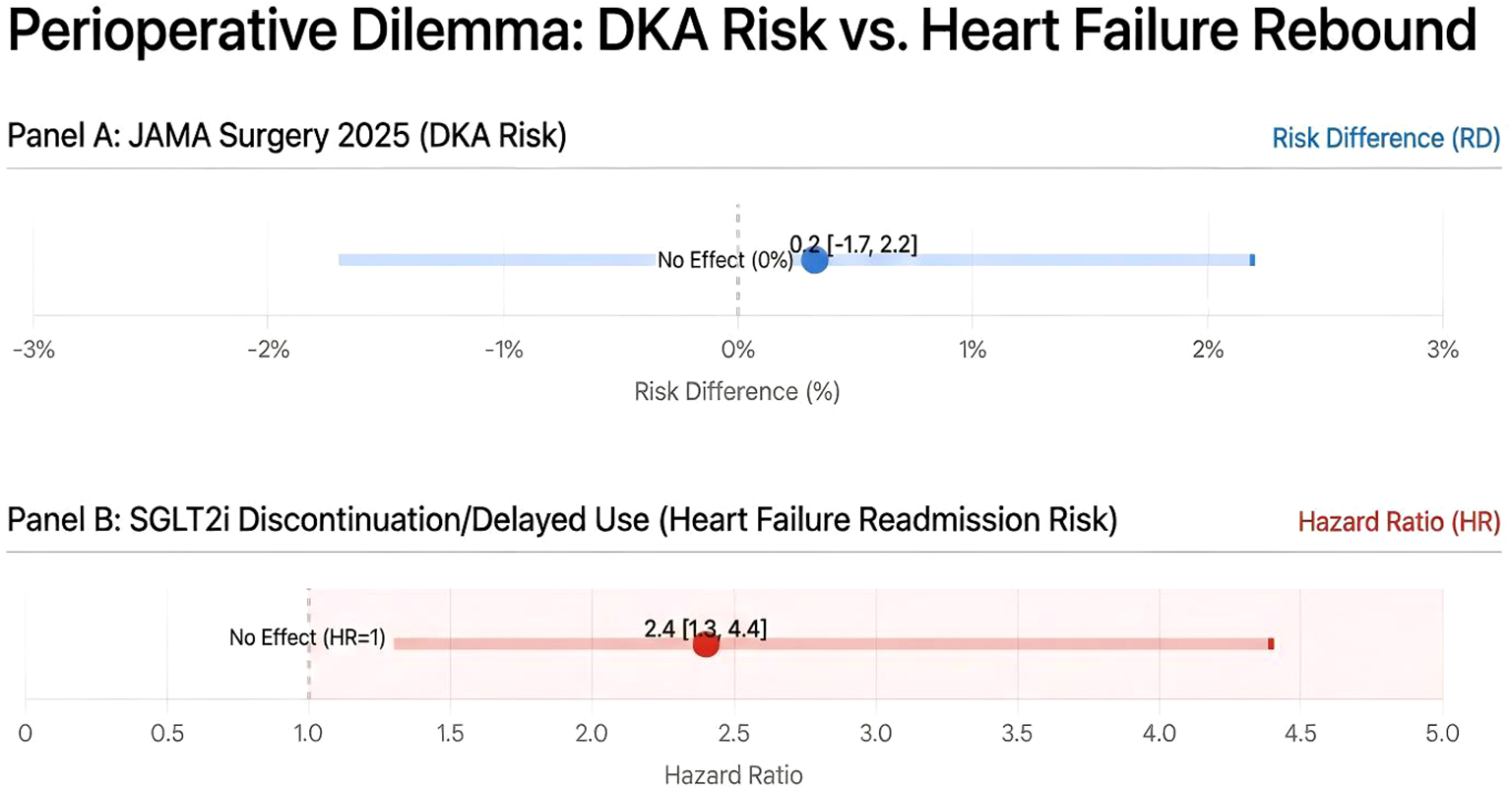
The multi-organ crosstalk of euDKA.

This comprehensive model explains why simple insulin replacement often fails to reverse the process if dehydration (the AVP driver) is not corrected, and why the condition can progress so rapidly in fasting patients (**Figure 1**).

This intricate multi-organ crosstalk provides a physiological lens through which to interpret the conflicting clinical evidence (6). The high-risk scenarios reported in the anesthesia literature—such as cardiac surgery with cardiopulmonary bypass or elective procedures with prolonged fasting—precisely create the conditions for maximal AVP secretion and SMCT upregulation (6). In contrast, the lower-risk profile observed in some emergency surgeries may reflect a shorter duration of these metabolic triggers (22). Thus, the mechanisms not only explain the ‘how’ of euDKA but also help predict the ‘when’ (23).

## 3 The clinical evidence crisis: divergent data in 2025

While the physiological mechanisms have become clearer, the clinical data governing perioperative management has entered a state of equipoise. The years 2024 and 2025 have witnessed the publication of conflicting datasets: large-scale retrospective analyses suggesting safety, contrasted against high-fidelity case series indicating severe risk.

### 3.1 The reassuring signal: JAMA surgery

A landmark study by Dixit and colleagues, published in JAMA Surgery in early 2025, has challenged the dogma of strictly withholding SGLT2 inhibitors (24).

- Study Design: The authors utilized a “natural experiment” methodology. They analyzed a nationwide cohort of over 34,000 patients with type 2 diabetes who underwent emergency surgery (defined as surgery occurring on the same day or within 2 days of an emergency department admission). The premise was that patients undergoing emergency surgery (e.g., appendectomy, acute cholecystectomy) would be unable to adhere to the FDA-recommended 3-day discontinuation period. Thus, this group served as a proxy for “non-withholding.”
- Results:
  - Unadjusted Incidence: The raw rate of postoperative DKA was 4.9% in the SGLT2i group versus 3.5% in the unexposed group.
  - Adjusted Analysis: Using targeted maximum likelihood estimation (TMLE) to adjust for confounders (HbA1c, insulin use, comorbidities), the study found no statistically significant association between preoperative SGLT2i use and postoperative DKA.
  - Average Treatment Effect (ATE): The ATE was 0.2% (95% CI, -1.7% to 2.2%), crossing the null value.
- Author’s Conclusion: The findings suggest that the current guidance to withhold SGLT2 inhibitors for 3–4 days may be overly conservative and could be “liberalized” to prevent the risks associated with medication disruption.

#### 3.1.1 Critique and limitations

While the JAMA Surgery study is methodologically rigorous, several critical limitations preclude utilizing it as the sole basis for changing practice:

##### 31.1.1.1 Coding blind spots

The study relied on ICD-10 codes (e.g., E11.1 for DKA). It is well-documented that euDKA is frequently undercoded. Because glucose levels are normal, clinicians often code these events as “metabolic acidosis” (E87.2) or “dehydration” rather than DKA. Although the authors attempted to include acidosis codes, the sensitivity of administrative data for this specific entity is likely low (24).

##### 3.1.1.2 Physiological differences

Emergency surgery differs from elective surgery. Emergency patients may be acutely stressed, but they often lack the prolonged “starvation” period (e.g., 24-hour liquid diet + bowel prep + NPO) typical of major elective colorectal or bariatric surgery. The duration of fasting is a key ketogenic trigger that might be less prominent in short-stay emergency cases (25).

### 3.2 The cautionary signal: BMJ open and anesthesia literature

In contrast to the reassuring administrative data, the anesthesia and intensive care literature continues to report clusters of severe euDKA. A 2025 systematic review and case series published in BMJ Open specifically examined outcomes in cardiac surgery (26).

- The Cardiac Cluster: The study identified 15 cases of euDKA in patients undergoing coronary artery bypass grafting (CABG) or valve procedures.
- Key Findings:
  - Trigger: The combination of cardiopulmonary bypass (which induces a systemic inflammatory response) and prolonged fasting was a potent trigger.
  - Morbidity: While mortality was rare (due to eventual recognition and treatment), the morbidity was substantial. Patients with euDKA had a median increase in ICU length of stay of 3 days compared to controls (26).
- The “Black Swan” Argument: Anesthesiologists argue that while the incidence might be statistically low (supporting the JAMA data), the consequence is catastrophic. In elective surgery, where the goal is perfection and rapid recovery (ERAS), a preventable metabolic crisis that adds 3 days to an ICU stay is considered an unacceptable “never event,” justifying the conservative withholding strategy.

### 3.3 The hidden cost of withdrawal: rebound heart failure

The debate is further complicated by the risks of stopping the drug. SGLT2 inhibitors are not just metabolic agents; they are hemodynamic stabilizers. They provide osmotic diuresis and reduce preload. Abrupt withdrawal in a patient with Heart Failure (HFrEF) can precipitate acute decompensation (27).

**Figure 2:**
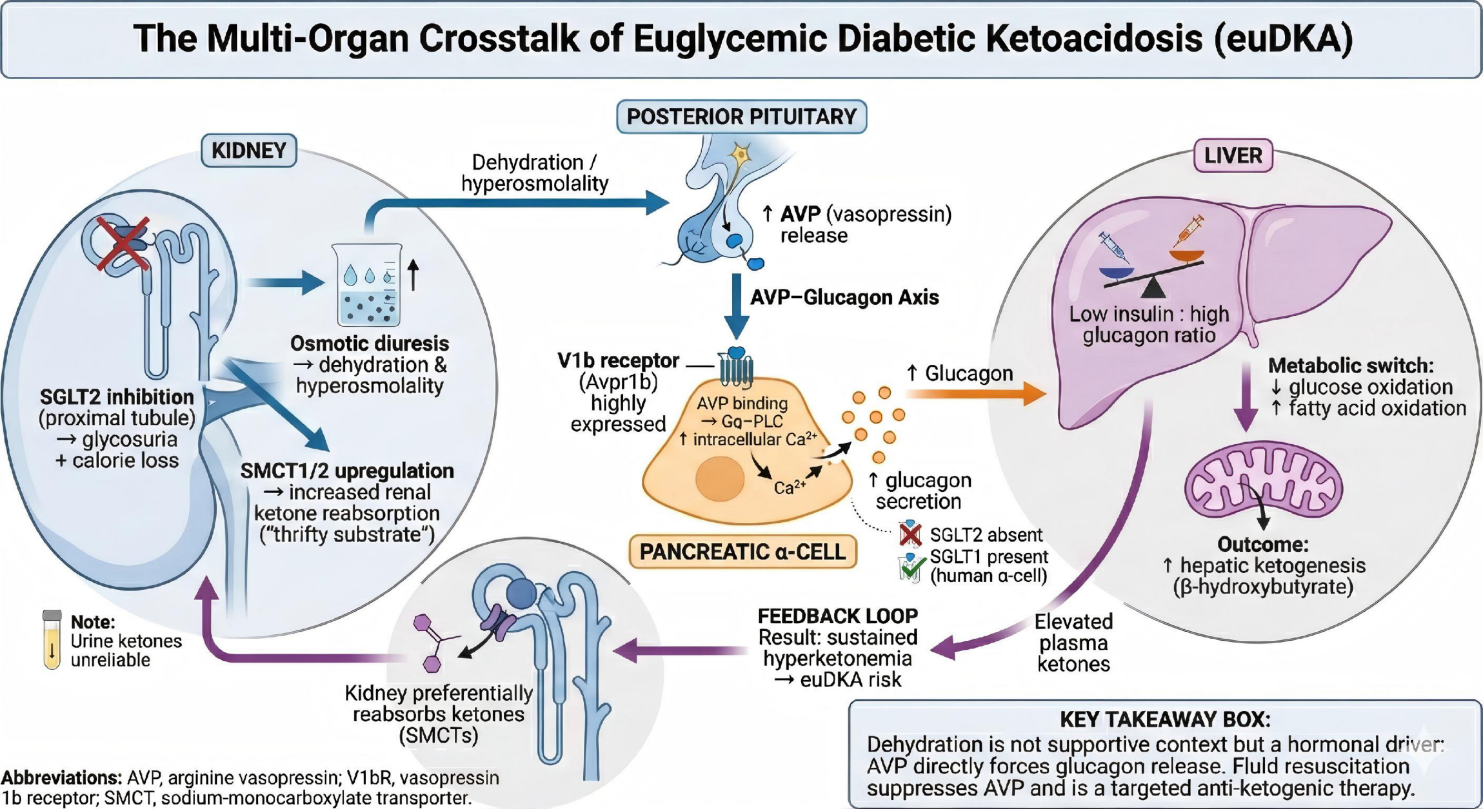
DKA risk vs. heart failure rebound.

- The Rebound Phenomenon: Cessation leads to the loss of natriuresis and a potential “rebound” increase in sympathetic tone.
- Readmission Data: Recent analyses indicate that discontinuing (or delaying the initiation of) SGLT2 inhibitors is associated with a significantly higher risk of cardiovascular rehospitalization. Specifically, data from BJCardio (2024) indicates a Hazard Ratio (HR) for cardiovascular rehospitalization of 2.40 (95% CI 1.31-4.41) in patients where SGLT2i therapy was not initiated/continued during admission compared to those who received it (28) (**Figure 2**).
- Implication: The surgeon’s fear of DKA (a rare event) must be balanced against the cardiologist’s fear of acute pulmonary edema (a common event). A 3-day preoperative stop, followed by a 3–4 day postoperative hold (due to ileus), results in a week-long hiatus in heart failure therapy, which is mechanistically sufficient to cause volume overload (29).

## 4 Comparative analysis of international guidelines

The divergence in clinical data has resulted in a heterogeneous regulatory landscape. While the FDA adheres to a pharmacokinetic-based “washout” model, other societies have adopted more pragmatic approaches based on clinical risk factors.

### 4.1 Comparison table

The following table synthesizes the current recommendations from major regulatory and professional bodies as of early 2025 (**Table 1**).

**Table 1:** Comparison of guidelines for preoperative discontinuation of SGLT2 inhibitors.

Guideline body	Region	Recommendation for preoperative cessation	Rationale & nuances	Reference
FDA (Product Label)	USA	3 days (Canagliflozin, Dapagliflozin, Empagliflozin) 4 days (Ertugliflozin)	Based strictly on drug half-life (12 h) to ensure 5 half-lives washout. Aims for complete elimination of the drug.	(30)
EMA (PRAC)	Europe	Stop during major surgery or acute illness.	Less prescriptive on “days” in the summary of product characteristics (SmPC), focusing more on clinical context. Emphasizes immediate cessation if ketosis is suspected.	(31)
ADA (Standards of Care)	USA	3–4 days before surgery.	Aligns with FDA guidance. Explicitly recommends resuming only when oral intake is normal.	(32)
ANZCA/ADS	Australia/NZ	3 days (2 days prior + day of surgery).	Promotes the “SSTOP” mnemonic. Highlights the importance of blood ketone testing if the patient is unwell.	(33)
JBDS-IP	UK	Variable (Often 3–4 days).	Individual Trust protocols vary. JBDS guidelines emphasize omission on the day of surgery and the day prior as a minimum, extending for high-risk procedures.	(34)
CPOC	UK	Day before + Day of surgery (Shorter window).	Acknowledges the risk of prolonged cessation (poor glycemic control, HF risk). Advises a shorter hold coupled with enhanced vigilance (daily ketones).	(35)

### 4.2 Synthesis of Differences: Pharmacokinetics vs. Pragmatism

- The Pharmacokinetic Argument (FDA/ADA): The FDA’s 3–4 day rule is derived from the half-life of SGLT2 inhibitors (typically 11–13 hours for dapagliflozin/empagliflozin) (6). Five half-lives are required to clear >95% of the drug. This strategy prioritizes the absolute prevention of drug-induced glycosuria during surgery (36).
- The Pragmatic Argument (CPOC): The UK’s CPOC and some anesthesia groups argue that a 24–48 hour stop significantly reduces plasma levels (by ~75-80%), which may be sufficient to lower DKA risk if combined with adequate hydration and carbohydrate supply. This approach prioritizes minimizing the “time off therapy” to protect the heart and kidneys (35).

## 5 Strategic perioperative management

**Figure 3:**
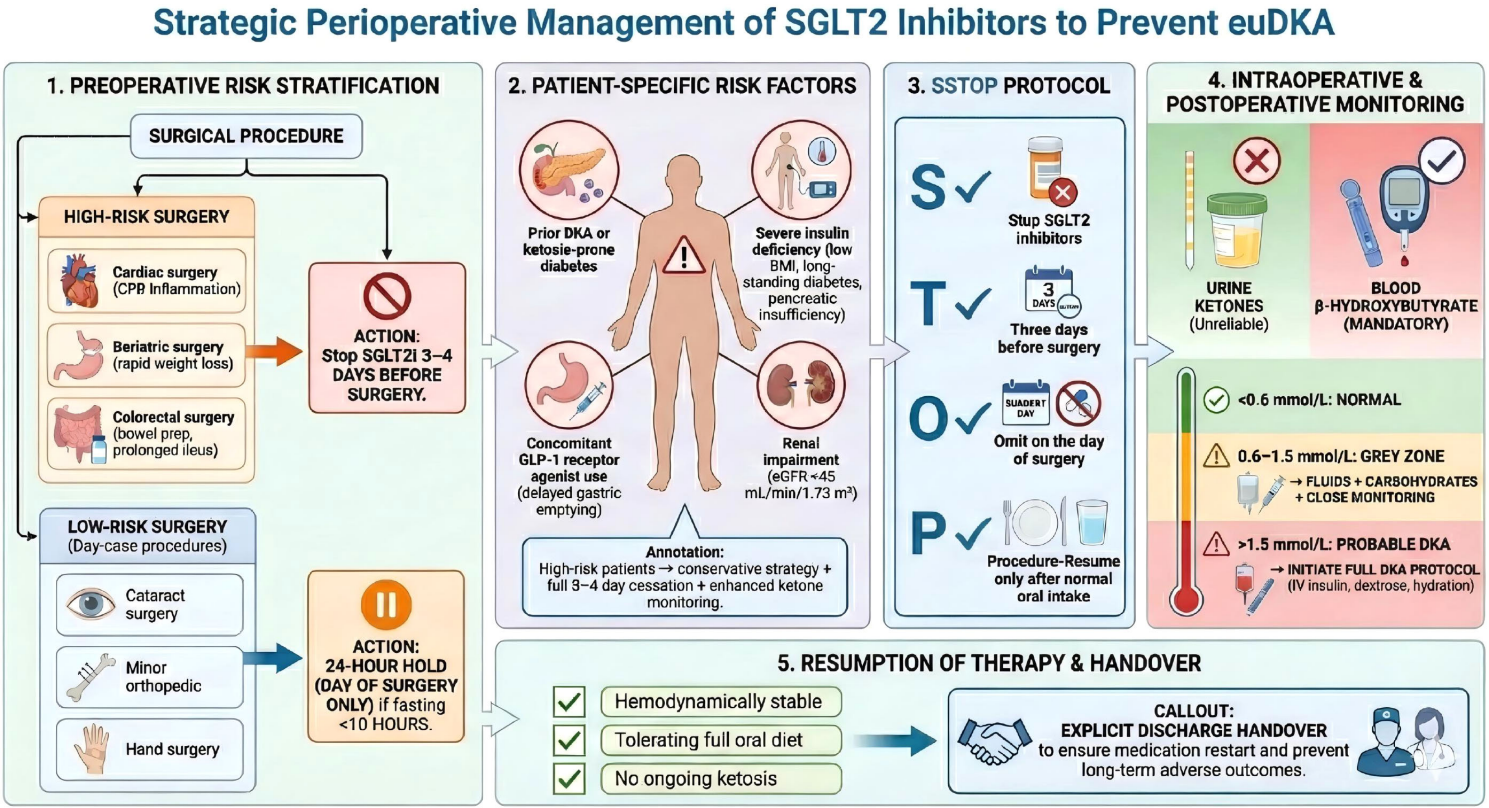
Strategic perioperative management.

Moving from guidelines to the bedside requires a structured, adaptable approach. The era of the simple “pocket card” checklist is ending, replaced by professional decision trees that integrate surgical risk, patient indication, and monitoring capabilities (**Figure 3**).

Notably, the mandate for hydration must be balanced against the risk of volume overload, particularly in patients with heart failure (HFrEF/HFpEF) or advanced chronic kidney disease (CKD) (37). In these high-risk individuals, ‘aggressive’ hydration is contraindicated. Instead, the goal shifts to optimized volume status (15). This often necessitates pre-operative consultation with cardiology or nephrology to establish a tailored fluid plan that ensures euvolemia—sufficient to suppress AVP-mediated hyperglucagonemia without precipitating pulmonary edema or worsening renal function (19). The principle remains: correcting dehydration is anti-ketogenic, but it must be achieved safely (23).

### 5.1 Preoperative risk stratification

The decision to withhold should be personalized based on the procedure’s ketogenic potential.

- High-Risk Procedures: Cardiac surgery (CPB inflammation), Bariatric surgery (rapid weight loss), Colorectal surgery (bowel prep + prolonged ileus). Action: Adhere to the 3–4 day rule.
- Low-Risk Procedures: Day-case surgery (e.g., cataract, minor orthopedic, hand surgery) where the patient returns to oral intake within hours. Action: A 24-hour hold (stopping on the day of surgery) is likely sufficient, provided the patient is not fasting for >10 hours.

### 5.2 Patient-specific risk stratification

Beyond the surgical procedure, individual patient factors significantly modulate euDKA risk. Key high-risk characteristics include:

- A history of prior DKA or ketosis-prone diabetes:Indicates an underlying predisposition to ketogenesis (38).
- Severe insulin deficiency:Often inferred from a low BMI, long-standing diabetes with glucopenia, or a history of pancreatic insufficiency (38).
- Concurrent use of glucagon-like peptide-1 receptor agonists (GLP-1 RAs): These agents can delay gastric emptying, potentially prolonging the fasting state and compounding the ketogenic drive (39).
- Baseline renal impairment (e.g., eGFR < 45 mL/min/1.73m²):Alters drug clearance and may exacerbate the metabolic disturbances of surgery (40).

Patients exhibiting these features warrant the most conservative management approach, including adherence to the full 3–4 day preoperative drug cessation and heightened perioperative ketone monitoring, even for intermediate-risk procedures (40).

### 5.3 The “SSTOP” Protocol

The “SSTOP” mnemonic remains the gold standard for patient education and pre-assessment nursing teams (41):

- Stop SGLT2 inhibitors.
- Three days before surgery (or 2 days prior + day of procedure).
- Omit on the day of surgery.
- Procedure (Resume only when eating/drinking normally).

### 5.4 Intraoperative and postoperative monitoring: the ketone imperative

The most critical safety net is monitoring.

- Blood vs. Urine: Urine ketone testing is unreliable in SGLT2i-treated patients. As discussed in Section 2.3, the upregulation of renal SMCT transporters increases ketone reabsorption, potentially leading to a “false negative” urine test even in the presence of significant ketonemia.
- Mandate: Capillary blood ketone (β-hydroxybutyrate) testing is mandatory for any patient on SGLT2 inhibitors who presents with nausea, vomiting, abdominal pain, or unexplained metabolic acidosis.
- Thresholds:
  - < 0.6 mmol/L: Normal.
  - 0.6 - 1.5 mmol/L: The “Grey Zone”. Action: This is not normal in the context of SGLT2i use. Administer fluids (reduce AVP), provide carbohydrates (suppress glucagon), and monitor hourly. Do not discharge if in this zone.
  - 1.5 mmol/L: Probable DKA. Action: Initiate full DKA protocol (IV Insulin + Dextrose + Aggressive Hydration). Do not wait for pH to drop.

### 5.5 Resumption of therapy

Restarting SGLT2 inhibitors is as critical as stopping them. Premature resumption (before oral intake is established) risks recurrent DKA. Late resumption risks heart failure decompensation.

- Criteria for Resumption: Therapy should be restarted only when the patient is hemodynamically stable, is tolerating a full oral diet, and has no evidence of ongoing ketosis (37). For patients requiring intravenous inotropes or vasopressors, resumption should be deferred until such support is no longer needed for indications related to the metabolic state(e.g., shock secondary to DKA). However, in cases where vasopressor support is for unrelated reasons (e.g., post-cardiac surgery vasoplegia) and the patient is metabolically stable, eating, and off insulin infusions, consultation with the critical care and cardiology teams is advised to weigh the benefits of restarting heart failure therapy against a low perceived risk of recurrent euDKA (15, 37).
- Handover: Explicit instructions must be given to the discharge team or GP to restart the medication, as “held” medications are frequently lost in transition, leading to long-term poor outcomes.

## 6 Conclusion and future directions

The perioperative management of SGLT2 inhibitors represents a dynamic intersection of endocrinology, nephrology, and anesthesiology. What began as a simple protocol of “holding a sugar pill” has evolved into a complex physiological challenge requiring a deep understanding of multi-organ crosstalk.

The mechanistic landscape has been redefined by the discovery of the AVP-V1b receptor axis and the renal SMCT “thrifty substrate” response. These pathways explain why dehydration is not merely a hemodynamic issue but a potent endocrine trigger for ketosis in these patients. Clinically, we stand at a crossroads. The 2025 JAMA Surgery data provides a reassuring population-level signal that the absolute risk of DKA in “non-optimized” emergency patients is low. However, the anesthesia literature reminds us that “low probability” does not mean “low consequence.” The morbidity of missed euDKA is severe, and the deceptive nature of the condition—normal glucose, negative urine ketones—makes vigilance paramount.

Recommendations for the Modern Clinician:

1. Adopt a Risk-Stratified Protocol: Move away from blanket “one-size-fits-all” cessation. Differentiate between major metabolic stress surgeries (stop 3–4 days) and minor procedures (stop 24 hours).
2. Target the Mechanism: Recognize that hydration is an endocrine therapy in this context. Aggressive preoperative hydration (where cardiac status permits) dampens the AVP signal and suppresses the ketogenic drive.
3. Upgrade Monitoring: Abandon urine ketone testing for diagnosis. Implement low-threshold capillary blood ketone testing for any symptomatic patient and treat the “grey zone” (0.6-1.5 mmol/L) with active carbohydrate/fluid management.
4. Balance the Risks: For high-risk heart failure patients, minimize the duration of interruption and involve the cardiology team to prevent rebound decompensation.

As we move forward, prospective randomized trials specifically comparing “short-stop” vs “long-stop” strategies—measuring both metabolic safety and cardiovascular outcomes—will be essential to resolve the current equipoise and refine our guidelines. Until then, a physiology-based, vigilant approach remains our best defense against this silent metabolic threat.
